# Comprehensive Characterization of PVDF Nanofibers at Macro- and Nanolevel

**DOI:** 10.3390/polym14030593

**Published:** 2022-02-01

**Authors:** Tatiana Pisarenko, Nikola Papež, Dinara Sobola, Ştefan Ţălu, Klára Částková, Pavel Škarvada, Robert Macků, Erik Ščasnovič, Jaroslav Kaštyl

**Affiliations:** 1 Department of Physics, Faculty of Electrical Engineering and Communication, Brno University of Technology, Technická 2848/8, 61600 Brno, the Czech Republic; Tatiana.Pisarenko@vut.cz (T.P.); papez@vut.cz (N.P.); sobola@vut.cz (D.S.); skarvada@vut.cz (P.Š.); macku@vut.cz (R.M.); 2 Institute of Physics of Materials, Czech Academy of Sciences, Žižkova 22, 61662 Brno, the Czech Republic; 3 Department of Inorganic Chemistry and Chemical Ecology, Dagestan State University, St. M. Gadjieva 43-a, 367015 Makhachkala, Russia; 4 Directorate of Research, Development and Innovation Management (DMCDI), Technical University of Cluj-Napoca, Constantin Daicoviciu Street, No. 15, 400020 Cluj-Napoca, Romania; 5 Central European Institute of Technology, Purkyňova 656/123, 61200 Brno, the Czech Republic; klara.castkova@ceitec.vutbr.cz (K.Č.); 201286@vutbr.cz (E.Š.); jaroslav.kastyl@ceitec.vutbr.cz (J.K.); 6 Department of Ceramics and Polymers, Faculty of Mechanical Engineering, Brno University of Technology, Technická 2896/2, 61600 Brno, the Czech Republic

**Keywords:** AFM, core-shell, DSC, electrostatic spinning, FIB, FTIR, hollow, hydrophobic, hydrophilic, nanofibers, PFM, PVDF, SEM, STEM, Raman spectroscopy, XPS

## Abstract

This study is focused on the characterization and investigation of polyvinylidene fluoride (PVDF) nanofibers from the point of view of macro- and nanometer level. The fibers were produced using electrostatic spinning process in air. Two types of fibers were produced since the collector speed (300 rpm and 2000 rpm) differed as the only one processing parameter. Differences in fiber’s properties were studied by scanning electron microscopy (SEM) with cross-sections observation utilizing focused ion beam (FIB). The phase composition was determined by Fourier-transform infrared spectroscopy (FTIR) and Raman spectroscopy. The crystallinity was determined by differential scanning calorimetry (DSC), and chemical analysis of fiber’s surfaces and bonding states were studied using X-ray photoelectron spectroscopy (XPS). Other methods, such as atomic force microscopy (AFM) and piezoelectric force microscopy (PFM), were employed to describe morphology and piezoelectric response of single fiber, respectively. Moreover, the wetting behavior (hydrophobicity or hydrophilicity) was also studied. It was found that collector speed significantly affects fibers alignment and wettability (directionally ordered fibers produced at 2000 rpm almost super-hydrophobic in comparison with disordered fibers spun at 300 rpm with hydrophilic behavior) as properties at macrolevel. However, it was confirmed that these differences at the macrolevel are closely connected and originate from nanolevel attributes. The study of single individual fibers revealed some protrusions on the fiber’s surface, and fibers spun at 300 rpm had a core-shell design, while fibers spun at 2000 rpm were hollow.

## 1. Introduction

Nowadays, nanofibers development, production and their properties characterization are of high interest among scientists [1,2,3,4,5,6], health doctors [4,7,8,9], and even between general public thanks to coronavirus pandemic situation (causing COVID-19 illness) and use of nanofibers in face masks [10,11] to prevent health of each individual. Another great use of fibers can be for energy harvesting or as sensors from fiber mats as the fibers are from piezoelectric material.

Piezoelectric materials can be polymer or ceramic and exhibit electrical polarization under mechanical stress. Therefore, mechanical energy is converted to electrical energy [12]. The advantage of piezoelectric polymers compared to piezoceramics is their high flexibility and ease processability [13]. Another advantage is their low toxicity and the possibility to produce fibers of different sizes and structures, from macro- to nanostructures. But, compared to piezoceramics, piezopolymers have significantly (difference in many orders of magnitude) lower values of piezoelectric parameters [5].

**Figure 1:**
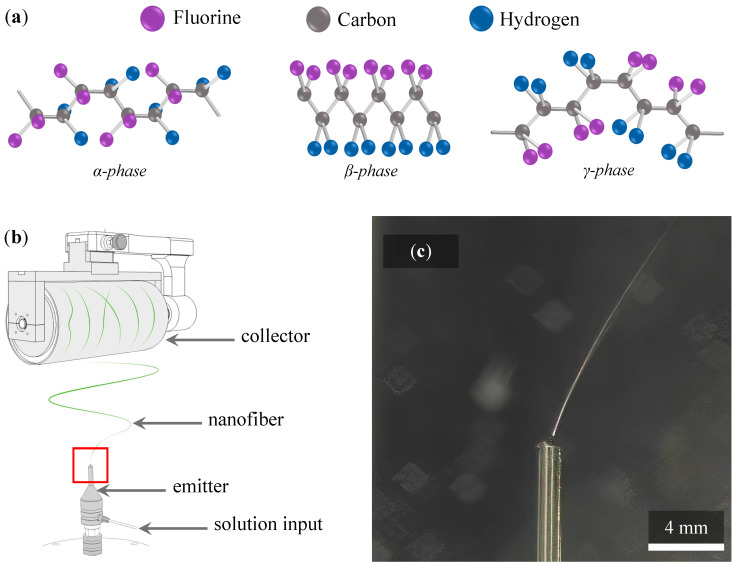
(**a**) Depiction of the chain conformation for the α-, β-, and γ-phase. (**b**) Illustration of electrostatic spinning using the single needle as the emitter, and the solid continual cylinder as the collector. The collected fiber is highlighted in green here (the actual color of the fiber depends on the fillers used—for example, the addition of carbon nanotubes is a dark black fiber, the addition of bismuth ferrite is a red fiber). (**c**) Detail of the polymer jet withdrawn from needle by applied high voltage.

In recent years, polyvinylidene fluoride (PVDF) has appeared to be a very promising material. It can be used as a non-degradable and biocompatible polymer for medical applications [14], energy harvesting [15,16], pressure sensors [17,18], electrically activated air filters [19], or viral particles filters [20] against to novel coronavirus [21], and to other similar viruses. PVDF is semi-crystalline fluoropolymer with excellent chemical stability, high mechanical strength, thermal stability, aging resistance and moreover, having molecules with large dipole moment perpendicular to the polymer chain. Compared to carbon and hydrogen, fluorine has a larger radius of the van der Waals effect, causing the dipole moment of the PVDF monomer [22]. It also depends on the configuration of the homopolymer chain. PVDF can exists in five phases: α, β, γ, δ, and ϵ. Phases α and ϵ are non-polar, the remaining phases β, γ, and δ are polar. Three of these phases illustrate Figure 1a. Thus, all polar phases exhibit piezoelectric properties, but most strongly contributing phase to piezoelectricity is the β-phase [5]. The phases differ by the configuration of the chain, the difference between phases α and β is usually presented in the literature as these phases are the most common because of electrospinning preparation procedure [5,23].

This leads to different electrical properties (depends on the crystal structure), which are used in many technical branches as mentioned elsewhere [24,25].

For this reason, it is highly desirable to aim attention to this material and focus on its unique properties, especially for electrical use [26]. Studying the production and characterization of PVDF, and thus observing effects of fiber mats and individual fibers especially essential for the knowledge development, and thus obtaining new possibilities of use in real life.

The schema of an electrospinning process, which is also used in this study, is shown in Figure 1b. The polymer solution (often delivered by a simple syringe pump) is connected to the emitter, which can be a needle with different tip and of various diameters on G (gauge) scale. The polymer solution is withdrawn from the needle tip when high voltage is applied, and polymer jet is formed and split in many fine thin streams creating so-called Taylor’s cone. The volatile compounds used in polymer solution evaporate during the fly phase as the fine polymer streams flow from the Taylor’s cone to the collector (here is the continuous cylinder used) at a controlled distance between needle tip and collector. Thus, fibers are formed at the end of the fly phase and immediately collected to form the fiber’s mat. The fibers are on the collector tensioned according to the speed at which the cylinder rotates. Fibers collected on the cylinder are also straightened, which results in the directionally ordered structure of fiber’s mat in one direction by default. The level of fiber’s order depends on the collector rotational speed. The real detail of the fine jet from needle tip, highlighted in Figure 1b by red square, is shown in Figure 1c. Please note that Taylor’s cone is captured in the Figure 1c, but it is difficult to recognize it as the cone is in micrometer scale and ultrahigh definition (UHD) camera with greater magnification, and the ultrafast shutter would be necessary.

Since electrospun fibers have tens to hundreds of nanometers in diameter, their analysis and characterization becomes challenging. As a result, very fine and precise techniques allowing to examine samples of only one single fiber are needed. In addition, only one method is insufficient for proper analysis of fiber’s structure and properties, so it is crucial to use several methods for reliable investigation and results [27]. A change of the only one processing parameter during fabrication does not have to affect only phase changes of nanofibers but also the fiber’s arrangement, size, shape, or their porosity, i.e., their structure completely. For example, their different arrangement may affect both the various piezoelectric properties within a single fiber and the strength, elasticity, or wettability of the entire sheet as a complete product [28]. Therefore, we conducted this study using all necessary fine analyses to show the difference in properties of fiber’s mats as well as of only one single fiber caused by the change of only one processing parameter, i.e., collector rotational speed. Thus, the novelty of this study lies in the characterization of whole fiber’s mats, but in addition, with great emphasis on characterization of individual fibers and the connection among differences and resulting findings are newly discussed.

As the PVDF fibers are piezoelectric, they should also be characterized for piezoelectric charge coefficient (often abbreviated as $d_{33}$ coefficient), relative permittivity, and dielectric loss as the most important piezoelectric properties. Taking into account that these analyzes have already been published [1,2,5], and also to keep this article clear, we have focused on other methods in this study and we do not mention piezoelectric properties here.

## 2. Material and Methods

### 2.1. Production of PVDF Nanofibers

The PVDF polymer (Sigma Aldrich, St. Louis, MO, USA) with molecular weight of 275,000 g/mol was dissolved in DMSO/AC (dimethylsulfoxide/acetone) solvent in a 7:3 volume ratio to create 20% polymer solution, based on our previous studies [1,5]. The PVDF fibers were electrospun using Contripro 4SPIN machine (Contipro, Dolní Dobrouč, the Czech Republic). This equipment was chosen due to the possibility of precise tuning of the processing parameters and thus as ideal for this study. The schema of spinning with this device can be seen in the illustration in Figure 1b.

The reason for choice of continuous rotating collector is that the thickness and thereby β-phase content in spun fibers can be precisely controlled. The fiber’s mats were produced at two different collector’s speeds, 300 rpm and 2000 rpm. Properties of fiber’s mats and individual fibers will be therefore compared based on the change of collector speed as the only one differing processing parameter in this work. The voltage between the collector and needle tip was set at 50 kV, and the collector was at distance of 20 cm from the emitter. The needle of only one diameter was 17 G (1.067 mm of inner nominal diameter) was used. No additional air and supporting heat were chosen. The solution flow from syringe to emitter was set to 18 μL/min. The complete processing parameters are summarized in Table 1.

### 2.2. Internal and External Structure of the Fiber

PVDF fibers were observed by scanning electron microscopes (SEM). Microscope with a focused ion beam (FIB), the Lyra3 (Tescan, Brno, the Czech Republic) was used for cross-section observation. The accelerating voltage was set to 5 kV for SEM observation. When cutting, the FIB high voltage was 30 kV, and for precise fiber separation, the current was only 50 pA. This is a small value for cutting, as the fiber had to be cut very finely to avoid defects. A thin layer of 20 nm thick carbon was also coated before observing due to fiber fixation and preventing charge accumulation.

SEM microscope with STEM detector—Helios NanoLab 660 (Thermo Fisher Scientific, Waltham, MA, USA), monitored the fiber composition using a high-angle annular dark-field imaging detector (HAADF). The accelerating voltage was set to 30 kV and the current 50 pA, which is a sufficient value for electrons that pass through the polymer fiber.

It is important to emphasize that the fibers were left uncoated for the purpose of STEM observation using HAADF, as they were observed longitudinally and thus, sputtered area could affect the observation. For this reason, it was necessary to make high-speed image accumulations. Fibers were placed on a gold grid here, and the observations took place very close to the conductive parts to allow the charge to dissipate. Only in this way was it possible to achieve relatively good image quality even though the fiber was not coated. The gold grid was placed on the aluminum substrate covering the collector so that all studied samples have identical parameters as they were produced at the same processing time.

### 2.3. Morphology and Piezoelectric Properties

Atomic force microscope (AFM) with piezoresponse force microscopy (PFM) mode (NTEGRA Prima, Moscow, Russia) with a tungsten carbide coated tip and bias voltage of −5 V to 5 V was used as characterization method for investigation of morphology and piezoelectric domains. The gold surface was used as a contact for characterization of PVDF fibers by electrical modes.

The samples were prepared by electrospinning method on the firm gold-coated silicon substrates with square dimension of 10 × 10 mm. The results after fabrication at collector speed of 300 rpm and 2000 rpm were compared. The area measured by microscope had dimensions of 10 × 10 mm. The measurements were performed in several sites and several times in a row to confirm the results. Only one fiber was characterized at a time. Visualization is performed in both two-dimensional and three-dimensional view. Scan velocity was from 8.04 μm/s to 13.94μm/s.

### 2.4. Elemental Fingerprint and Chemical Composition

Raman spectroscope alpha300 R (WITec, Ulm, Germany) was used to monitor the phase formation described. A green laser with a wavelength of 532 nm and with a power of 5 mW was used. The spectrum was accumulated 30× with an integration time of 7 s. The magnification of the lens was set to 100×. Due to the thickness of the laser beam incident on the sample’s surface, a single fiber is not possible to characterize. The diameter of fibers ranges from tens of nanometers to units of microns. The fibers should not be stressed during measurement with high laser power and long irradiation time. Otherwise, they may degrade and thus change the resulting spectra. Due to the inhomogeneous surface of the sample, the measurement was performed in several sites and several times in a row. The uneven surface and the characteristic shape of the fibers also affected the overall reflection of light and thus reduced the total signal intensity.

Transmission experiment was performed by Fourier infrared spectrometer Vertex 80v (Bruker, Billerica, MA, USA), also for phase investigations [29]. The spectral range 4000 cm^−1^ to 400 cm^−1^ was measured. From this range, area of interest was selected. The measurement took place in a vacuum chamber. A pre-measured background was subtracted from the resulting spectra, and a baseline correction was performed. The amount of phase β + γ, β, and α was calculated from FTIR measurements. For example composition of electrically active β + γ phase is determined from Equation (1) [5,7]:

(1) $$F\left(\beta +\gamma \right)=\frac{A_{\beta ,\gamma }}{1.26A_{\alpha }+A_{\beta ,\gamma }},$$

where $A_{\beta ,\gamma }$ and $A_{\alpha }$ are the absorbance values at 763 cm^−1^ and 840 cm^−1^.

Observations of the structure and electrical properties were also supplemented by AXIS Supra (Kratos Analytical, Manchester, UK) X-ray photoelectron spectroscope to determine the fiber’s elemental composition and its chemical states. Wide spectra and high-resolution bands were measured. The emission current of 15 mA was the same for both types of measurements. The binding energy of the wide spectrum was measured in the range of 1200 eV to 0 eV.

### 2.5. Wettability of a Solid Surface

The See System (Advex Instruments, Brno, the Czech Republic)—a computer-based instrument with a mechanical table and a camera for sensing a drop of liquid on the measured sample was used to measure the contact angle and wettability of the samples. The captured images allow matching of the profile of the droplet and the arc. The software creates and calculates the angle formed by the substrate of the drop and the tangent of the arc at the point of intersection with this substrate. Based on the records of continuous measurement, it is possible to demonstrate the time evaluation of the contact angle [30]. When a drop of fluid is in contact with a flat solid surface, a final contact angle is formed, and the shape of the drop depends on the relative magnitudes of the molecular forces that exist inside the liquid (cohesive) and between the liquid and the solid surface (adhesive).

The sample was cut in the form of a thin strip on which 10 droplets of demineralized water were gradually applied. The measurement is affected by significant deviations due to random and systematic errors. Therefore, it is advisable to measure the sample in several places and several times to eliminate these errors. Each droplet of water had a volume of exactly 3 μm. After applying the droplet on the sample, 5 s were allowed to stabilize. This exact time was measured thanks to the continuous imaging of the computer application by the camera. After this time, the contact angle of the droplet was recorded. Measurement was performed 10× for one type of sample. All results were averaged. The results were classified as either hydrophilic < 90°, hydrophobic > 90°, highly hydrophobic > 120°, or superhydrophobic > 150°.

### 2.6. Determination of Polymer Crystallinity

Phase transitions known as crystallization or melting were determined by DSC 204 F1 (NETZSCH, Selb, Germany), the instrument used for differential scanning calorimetry. The crystallinity percentage was calculated and compared for PVDF nanofibers with different fabrication parameters (solution substances for both specimen types were identical). The aim was to determine whether the tension of the fiber during higher collector speeds directly affects its crystallinity. An argon flux was 20 mL/min during a heating rate of 10 °C/min. A temperature range was chosen from 25 °C to 200 °C, but the final characteristic was specified from 40 °C to 190 °C to highlight the essential parts of the plot.

## 3. Results and Discussion

The results obtained from measurements using several methods stated in Material and methods—Section 2 are described below. These are measurements by electron microscopy, including transmission observation with a scanning transmission electron microscopy (STEM) detector and observation of the fiber in cross-section created by focused ion beam (FIB). Examination of the piezoelectric response by PFM method was also critical, given that this is a unique feature that makes PVDF attractive. Spectral analysis was performed by Raman spectroscopy, Fourier-transform infrared spectroscopy (FTIR), and X-ray photoelectron spectroscopy (XPS). The wettability and contact angle of water were also measured on the samples. The produced PVDF nanofibers are characterized both in terms of material and structural, as well as in terms of elemental composition. The most measurements were performed on two different types of samples—at different collector speeds of 300 rpm and 2000 rpm of electrospinning instrument [31]. Despite the change of one production parameter, different results can be observed for all methods.

### 3.1. Examination of the Structure by Scanning Electron Microscopy

**Figure 2:**
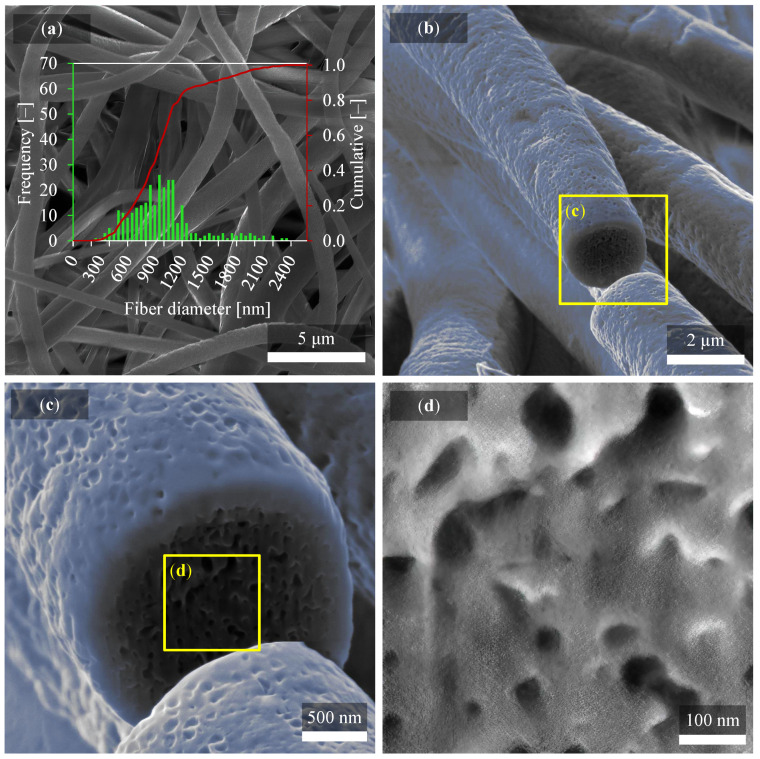
(**a**) SEM image of PVDF fibers prepared at 300 rpm with (**b**) detail of PVDF nanofiber in cross-section and its porous structure after fabrication with 300 rpm of collector speed. All four figures describe the same fiber structure. The cross-section highlighted in (**b**) is more in detail shown in (**c**) and in maximized detail in (**d**).

**Figure 3:**
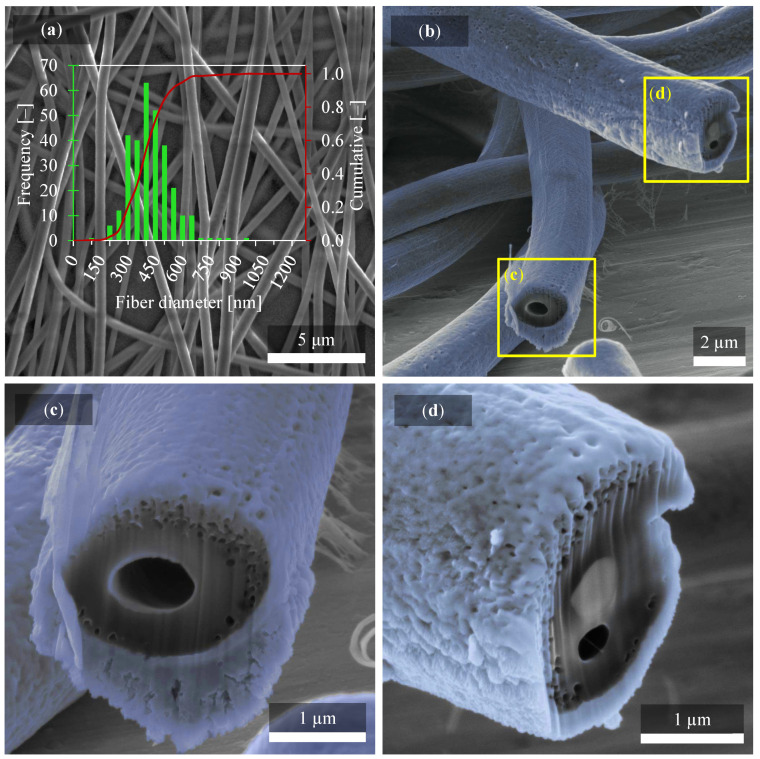
(**a**) SEM image of PVDF fibers prepared at 2000 rpm with (**b**) detail of PVDF nanofiber in cross-section and its porous structure after fabrication with 2000 rpm of collector speed. All four figures describe the same fiber structure. The cross-section focused from (**b**) to the detailed part of the individual fibers (**c**,**d**). Please note that the range of x-axis in (**a**) is half of the range of histogram used in Figure 2a.

From the results below, several findings affecting the material properties are evident. In Figure 2, nanofibers are observed by SEM imaging with clearly different thicknesses compared in Figure 3. It can be seen that at different collector speeds, the arrangement and shape of the nanofibers are very different. Thus, a higher collector speed causes thinning of the fibers [29] since no other fabrication parameters were changed. However, it can certainly be argued that the production of nanofibers at higher collector speed causes their elongation and subsequent thinning. These differences affect the outcome of piezoelectric behavior. Interestingly, in Figure 3a fiber diameters are relatively similar compared to Figure 2a, where fibers vary from 230 nm to 2.33 μm.

A 95% confidence interval using Student’s t-distribution was calculated from 300 values measured on fibers in SEM micrographs and from standard deviation over these values. The average fiber diameter was (966 ± 44) nm and (395 ± 13) nm for fiber mats produced at 300 rpm and 2000 rpm, respectively.

Disordered and uniformly ordered fibers alignment can then have a significant effect on many material properties. On closer examination, it was focused directly on the characterization of one fiber by SEM microscopy, as described in Figure 2b–d and Figure 3b–d. Since the fibers exhibit considerable movement when irradiated with the electron beam and there is charging and the low possibility of observation, it was necessary to perform a coating. A carbon was fully sufficient for this purpose. Standard thicknesses for observing non-conductive materials with an electron microscope are about 10 nm or less. However, in our case, it was necessary to use thicker layers with a value of 20 nm, since, despite the 10 nm layer, there was still movement of nanofibers, which means a relatively high electroactivity.

The gradual view field, where a yellow rectangle bounds the magnification area in Figure 2b,c, captures the nanofiber down to the most significant detail. Figure 2b shows some of the fibers which were focused on. One of them has been chosen and very finely cut by the FIB with a current of 50 pA. The melting point of polyvinylidene fluoride is 177 °C, but even much lower temperatures already affect its structure. Such a precise cut allowed no melting by beam and other deformation to occur, as seen in Figure 2c. Deformation of the fiber would distort the complete results, and the final image would not be beneficial. Figure 2b,c also show colored parts. These colored parts represent the carbon, i.e., a thin protective layer, thanks to which the fiber was coated and fixed against movement. It was colored by blue from the differentiation and better orientation for the reader from the original PVDF material. The carbon coating is, therefore, not an original part of nanofibers. In Figure 2d, there is no coating, as can be seen. This is the focus of the fiber core itself, where a porous structure is visible. Such pores reach a diameter of about 5 nm to 50 nm, and their occurrence is in almost every fiber. Thus, it can be assumed that the strength of the fiber is directly affected by this porous structure and its other properties. Which is crucial, for example, for the tensile strength of the fiber.

Another very interesting phenomenon is shown in Figure 3, where again the fiber’s cross-section is performed similarly as in the previous case with a current of 50 pA. In the same way, it is distinguished here by the blue color carbon coating, which is applied only for the needs of observation under an electron microscope. The yellow rectangles are centered on the cross-section of the fibers, which are shown in Figure 3c,d. In both cases, there is a large gap inside the fiber. It can also be said about Figure 3c that the fiber is hollow inside. Particularly around this fiber core, the porosity no longer occurs as in the previous case in Figure 2. The occurrence of smaller holes/pores is rather along the edges [32].

**Figure 4:**
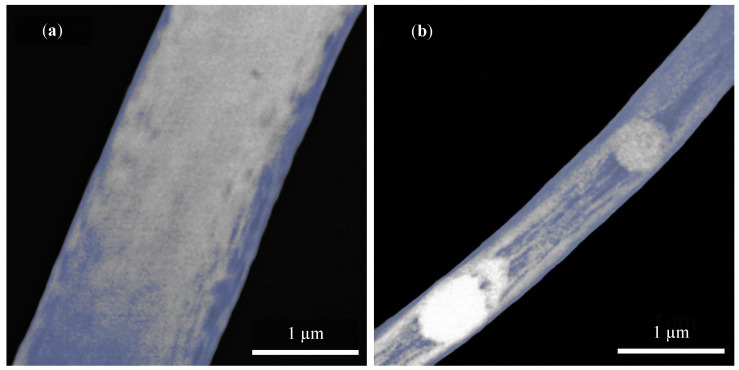
Internal fiber structure at collector speed (**a**) 300 rpm and (**b**) 2000 rpm observed by STEM detector and HAADF method. The accelerating voltage of 30 kV with the current of 50 pA and view field of 5 μm was used.

Figure 4a shows the fiber produced at 300 rpm and Figure 4b at 2000 rpm. The bluish tone emphasizes different fiber structures (not the carbon coating, as in previous figures). In addition to the variety in distinct fiber thickness, which is different at first glance, the fiber structure affects not only the strength but also the piezoelectric properties [1]. Interesting are defective spherical shape structures that occurred over almost the entire width of the fiber in Figure 4b. The origin of these shapes is unknown and may be caused, for example, by the collector speed, a dosing rate, or a solution concentration [1,33]. This phenomenon opens up further possibilities for research and testing of defects depending on manufacturing parameters.

### 3.2. Fibers Morphology and Electrical Parameters Obtained Using Atomic Force Microscopy

**Figure 5:**
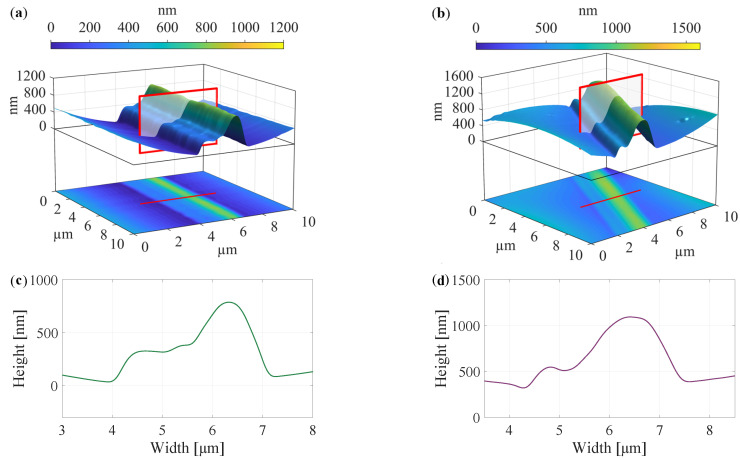
Morphological structure of PVDF nanofibers scanned by AFM with different collector speeds at (**a**) 300 rpm and (**b**) 2000 rpm, and their software-estimated cross-sections in (**c**) and (**d**), respectively.

The defective rounded structure, which is visible, for example, in Figure 3d by SEM was also scanned using AFM for the cases of both collector speeds in Figure 5. Morphology is captured in both three-dimensional and two-dimensional representations [34]. It was confirmed that fibers of imperfect shapes form at both speeds of 300 rpm (Figure 5a) and speeds of 2000 rpm (Figure 5b).

As a post-processing method, a cross-section was created using Matlab 2021b software. Microscope data were extracted and processed. As shown in Figure 5, red rectangles were drawn across each fiber in the three-dimensional graph and lines in the two-dimensional graph. These marks indicate a cut similar to that made by the FIB in Figure 2 and Figure 3. Of course, in this case, it is a software cut, which means that no formations can be seen in the fiber’s core. However, this method is handy if we need to know the exact morphology, more precisely, the profile of the fibers [35]. Since AFM has a much higher resolution than SEM, which was used in this work (better resolution can be achieved only with a TEM microscope), the possibility of obtaining very useful morphological information is achieved. In Figure 5c,d it can be seen, as already indicated, that the profile has a relatively similar shape in both cases of the electrospinning speed. It is also important to note the different ratio of *x*- and *y*-axis, which is intentionally different for fiber shape specifications. It can be stated that the shape of these fibers is not directly affected by the speed of the collector. On the contrary, it affects the thickness of the fibers, which has already been mentioned and is clearly evident from Figure 2a and Figure 3a, or Figure 4 [36].

**Figure 6:**
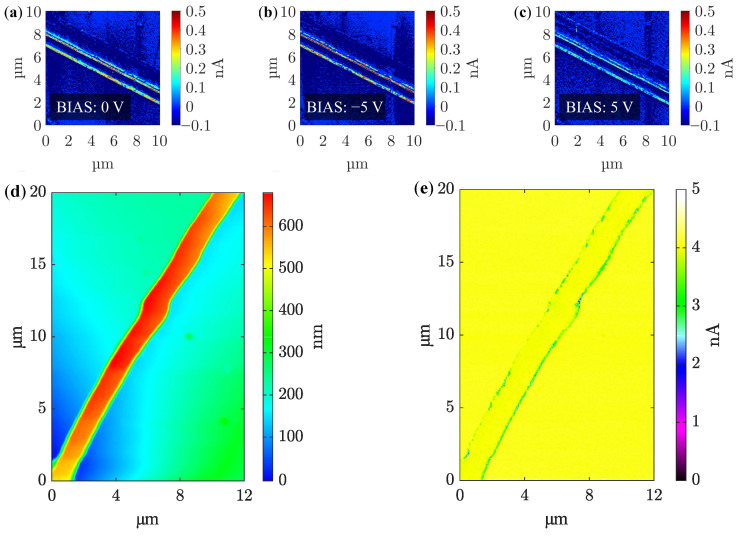
(**a**–**c**) Piezoelectric response of PVDF nanofiber with the imperfect circular shape at biasing of (**a**) 0 V, (**b**) −5 V, and (**c**) 5 V. (**d**) Measured fiber morphology and (**e**) electrical response of vertically oriented domains of single fiber by PFM method.

The same fiber measured by AFM microscope was also investigated by PFM method. The piezoelectric response of the fiber was confirmed. PFM method was performed for the fiber with a more decisive β-phase—at the collector drum speed of 2000 rpm (Figure 5b). The change in polarization is most visible along the edges of the fiber, described in Figure 6a–c. Here, the color of these protrusions changes from light green to deep red in the form of a thin line. The voltage was varied from −5 V to 5 V. It was verified that the shape of the fiber has a direct effect on its electrical properties and behavior. On the edges of the fiber, which was observed from its morphology in the previous Figure 5b, dipole changes due to the adjustment of bias voltage from −5 V to 5 V are noticeable. These dipole changes are significantly affected by hysteresis when returning to 0 V. From this point of view, it can be stated that nanofibers are functional with the piezoelectric response, but they are affected by its shape [31,37].

Another measurement using piezoresponse force microscopy monitors the exact morphology (Figure 6d) of the fiber and its electrical parameters (Figure 6e), which vary mainly along its perimeter up to 1 nA. In this case the presence of electrostatic interaction should be taken into account. By this reason, it is possible to follow only qualitative difference of the fibers appearance after applying large voltages. The response of vertically oriented domains is observed in Mag-signal [13].

### 3.3. Identification of PVDF Phases and Chemical States

**Figure 7:**
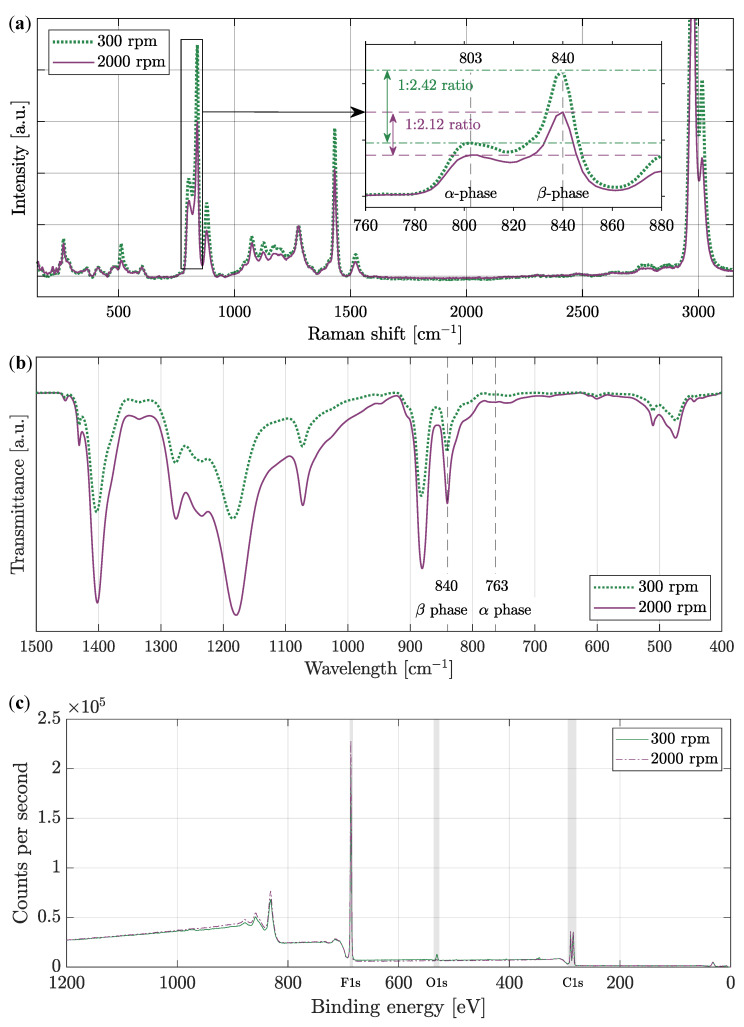
Nanofiber PVDF material investigated using (**a**) the Raman spectroscopy with focus to 760 cm^−1^ to 880 cm^−1^ region. (**b**) FTIR transmittance spectra with emerging phases α and β, indicated by a dashed line. (**c**) XPS wide spectra of PVDF nanofibers, for both types PVDF electrostatically spun at 300 rpm and 2000 rpm collector speed.

The Raman spectroscopy and FTIR were used to identify PVDF phases, and XPS analyses were done to determine chemical states. Typical Raman spectra of PVDF material in Figure 7a describes a molecular parameters in the range of 150 cm^−1^ to 3150 cm^−1^. Attention was focused on the spectrum in the range of 760 cm^−1^ to 880 cm^−1^, where α- and β-phase peaks are illustrated in detail and differences in their ratio are measured [38]. The differences in ratio on the two different types of samples are less noticeable in Raman spectroscopy, and therefore FTIR was used for a more precise comparison of the β-phase [39].

FTIR spectra in Figure 7b shows dependence of the transmittance on the wavelength of the whole sample between 1500 cm^−1^ to 400 cm^−1^. The β-phase is strongly visible at 840 cm^−1^ [40]. It is evident that the β-phase is higher for fibers produced at the collector speed of 2000 rpm than for fibers produced at a collector speed of 300 rpm. So, it can be concluded that the piezoelectric effect will show higher values for the fibers at the collector speed of 2000 rpm. The calculated β + γ/β/α phase content is for 300 rpm speed 90.31 wt%/77.71 wt%/9.69 wt%, and for 2000 rpm speed, it is 94.22 wt%/82.66 wt%/5.78 wt%. This calculations confirms the greater amount of β- and γ-phases in the sample spun at higher collector speeds.

The surface chemistry of a material to approx. depth of 10 nm was examined by measuring the kinetic energy of electrons using XPS. From the survey spectrum of PVDF polymer and wide energy range, certain significant peaks are observable, which indicate the presence of several important elements in Figure 7c. As it is already known from the chain conformations shown in Figure 1a, PVDF consists mainly of hydrogen H, fluorine F, and carbon C with chemical formula $-{\left(C_{2}H_{2}F_{2}\right)}_{n}-$. Also, it is essential to mention that hydrogen contains only one valence electron, and therefore it is not possible to detect it with the X-ray photoelectron spectroscopy method. However, oxygen can also be detected on the material’s surface. As already known, oxygen is found in almost every surface exposed to the atmosphere. Thus, in Figure 7c, peaks carrying important information are indicated by a gray stripe. The peaks in the region of C1s, O1s, and F1s, i.e., core-level emissions, are significant. The Figure 7c shows samples made at both 300 rpm and 2000 rpm.

**Figure 8:**
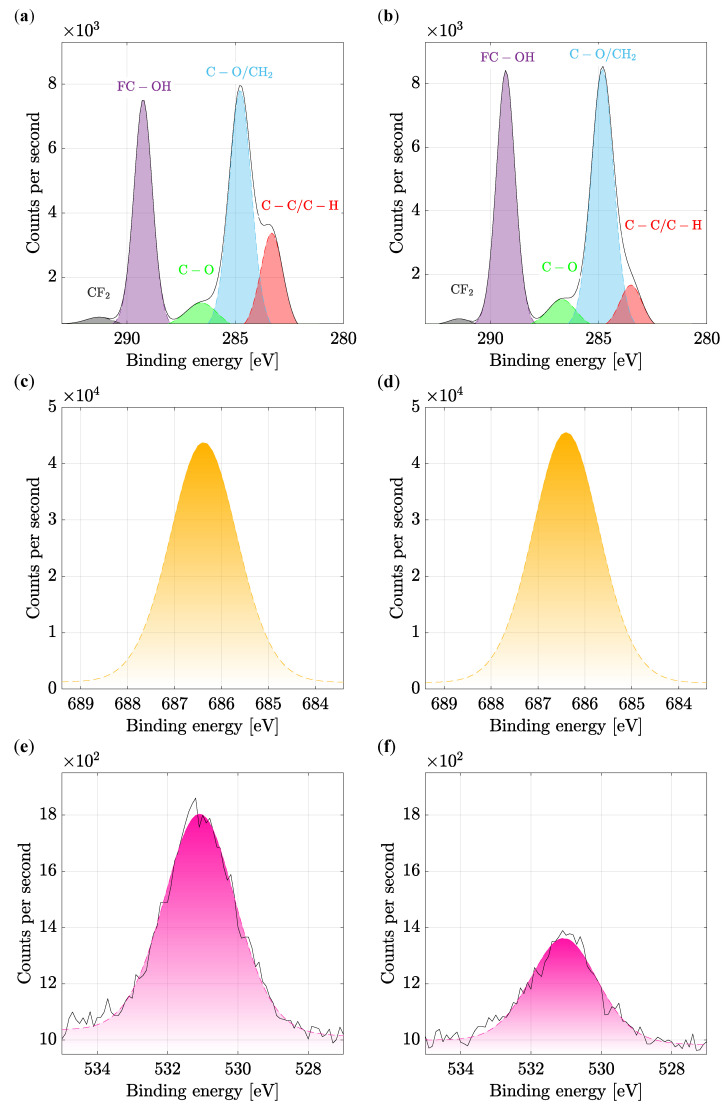
XPS spectra in (**a**,**b**) of the C1s high-resolution energy band’s expressed by an envelope, fitted and divided into individual peaks of certain bonds in the material [44]. A more substantial C−C/C−H binding (red peak) is recognised on the fiber produced at (**a**) 300 rpm compared to the fiber produced at (**b**) 2000 rpm. High-resolution energy band of F1s orbital electron is in (**c**,**d**). High-resolution energy band of O1s orbital electron is shown in (**e**,**f**). Please note that plots in (**a**,**c**), and (**e**) are depicted for PVDF fibers electrospun at 300 rpm collector speed, whereas plots in (**b**,**d**), and (**f**) are shown for nanofibers at 2000 rpm collector speed.

One of the most crucial spectra can be considered the carbon region. In addition to the sample components, it can also be adventitious carbon contamination, therefore, similarly to oxygen, it can occur more or less in many samples. However, in this case, the presence of carbon in the sample is desirable, as was written above. In the C1s region, there are several critical binding energies of chemical states that are characteristic for PVDF. A typical carbon fingerprint of PVDF is evident in Figure 8a,b. Occurring bonds are ${\mathrm{CF}}_{2}$ (gray), FC−OH (purple), C−O (green), $C-O/{\mathrm{CH}}_{2}$ (blue), and C−C/C−H (red). Since they cannot be directly identified from the measured spectrum, which delimits the bonds by the black line, it was necessary to use fitting, i.e., post-processing after the measurement, and to model them mathematically. From these results, particular bonds can already be successfully determined [41]. In the sample in Figure 8b, a change in the ratios of the FC−OH and $C-O/{\mathrm{CH}}_{2}$ bonds is observed, as well as a slight increase. On the contrary, a significant decrease occurred for C−C/C−H [42].

Minimal changes in Figure 8c,d are observed for fluorine. These changes are mainly due to minor differences between semi-ionic and covalent bonds, which means an insignificant crystalline phase shift. For a sample produced at 2000 rpm, this shift is then observable from the increase in binding energy.

From the O1s region in Figure 8e,f, oxygen can be recognized. Oxygen was introduced into the PVDF during production, where PVDF was exposed to air. In most cases, this region is composed of peaks with oxygen bounds to carbon and hydrogen. Due to the minimal occurrence, which can be compared in relation to the other regions in Figure 7c, the binding energy for the O1s region is very small. For this reason, the actual measured value without fitting (which is colored in purple) was also marked in figure with a black line. In other cases, nevertheless, the measured spectra are very detailed and accurate. Figure 8f shows a significant decrease in the oxygen bound to PVDF produced at a higher rate of 2000 rpm [43]. It is worth mentioning that both samples were produced immediately after each other and isolated, so the change in humidity has no significant effect here.

### 3.4. Influence of Differently Produced Nanofibers on Their Wettability

**Figure 9:**
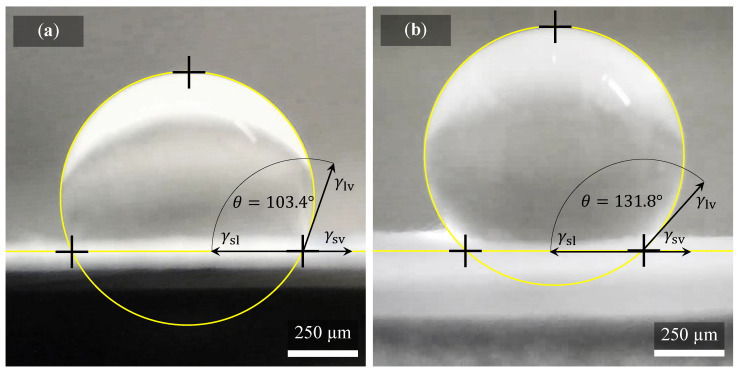
Hydrophobicity and hydrophilicity measurement. Each image shows 3 μL droplet on the specimens surface. Images show the droplet on surface of (**a**) disordered and (**b**) ordered fibers spun at 300 rpm and 2000 rpm, respectively. The different wettability of both structures is obvious.

Evaluation of hydrophobicity and hydrophilicity by measuring the contact angle of the droplet of demineralized water on the surface of the sample of PVDF nanofibers at two different collector drum speeds showed significant differences. Figure 9 shows two droplets of water. Each applied to a differently made specimen. The contours of the water droplets are marked by a yellow line, as well as the interface between the sample and another environment. The contact angle gripping the water drop with the sample in the form of black arrows is also drawn in the images for better orientation [45]. Quantities are defined in the Young equation, where $\gamma_{\mathrm{sv}}$ is the free solid-vapor interfacial energy, $\gamma_{\mathrm{sl}}$ is the free solid-liquid interfacial energy, and $\gamma_{\mathrm{lv}}$ is the liquid-vapor interfacial energy (surface tension). This balance can be expressed as $\gamma_{\mathrm{sv}}-\gamma_{\mathrm{sl}}=\gamma_{\mathrm{lv}}\;\cdot \;\cos\theta$.

It is clear from comparison of the droplets shape (Figure 9) that mechanical balance of droplets differs significantly. The droplet on sample surface in Figure 9a spun at 300 rpm exhibit contact angle of 103.4 ± 4.2° resulting as more hydrophilic than fiber spun at 2000 rpm (Figure 9b), showing the contact angle of 131.8 ± 2.9°. The enhanced contact angle of PVDF fibers spun at 2000 rpm can be sign of higher porosity on fiber surface [46], or it can be caused by higher surface roughness [47], or due to parallel alignment of the fibers [48]. But in general, samples where fibers are disordered as well as fibers ordered in one direction (spun at higher speed) can be considered as hydrophobic since in both cases, the contact angle of 90° determining the hydrophobicity of the material was exceeded. The droplet which almost approaching superhydrophobicity (contact angle of 150° [49]) is shown in Figure 9b.

### 3.5. Crystallization Behavior of PVDF Using Differential Scanning Calorimetry

**Figure 10:**
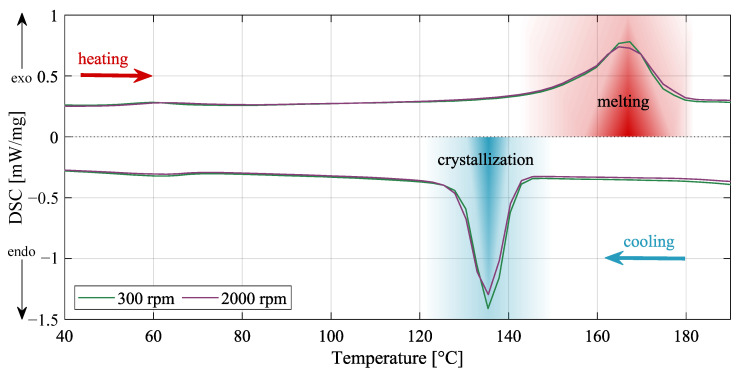
Differential scanning calorimetry (DSC) curves for both PVDF sample types. The differences in exothermic and endothermic peak regions of DSC curves are minimal.

Crystalline phases $X_{C}$ were calculated from Equation (2) [1]. Both samples achieved very similar crystallinity, which was expected due to the same type of solution and preparation, but slight deviations occurred. Specimen prepared on 300 rpm of collector speed reached 49.27% crystallinity, and for the 2000 rpm of collector speed, it was 50.55% crystallinity. According to

(2) $$X_{C}=\frac{\Delta H_{f}}{\Delta H_{f\varphi }^{*}}\;\cdot \;100,$$

where $\Delta H_{f\varphi }^{*}=104.7\;J/g$ is a heat of fusion of perfect crystalline PVDF, from the measured samples must also be calculated enthalpy of fusion $\Delta H_{f}$. Thus, to complete the equation, the specimen prepared on 300 rpm of collector speed reached of $\Delta H_{f}=51.58\;J/g$, and for the 2000 rpm of collector speed, it was $\Delta H_{f}=52.92\;J/g$ [1]. The exact behavior of the samples can be seen in Figure 10.

## 4. Conclusions

Polymeric PVDF nanofibers were successfully prepared in two types within a controlled electrospinning process using two collector speeds as the only one differing processing parameter. Structural, molecular, and electrical properties of PVDF nanofiber mats and individual single nanofibers were studied.

Fibers with average diameter of 970 nm and 400 nm at the rotating collector speed of 300 rpm and 2000 rpm, respectively, were obtained. Another substantial difference is the inhomogeneity and directional disorder of fibers in fibers mat spun at 300 rpm whereas sample spun at 2000 rpm resulted in directionally ordered fibers. Moreover, fibers spun at 300 rpm had core-shell morphology with pronounced porosity in the core and denser shell. Totally different morphology, hollow fibers, were obtained when rotating speed was 2000 rpm.

In the morphology analysis of a single fiber by atomic force microscopy, it was found that individual fibers contain some protrusions and other imperfections deviating from the ideal round shape without the presence of any powders or other fillers. The piezoelectric force microscopy revealed that the response of vertically oriented domains could be recorded mainly on these protrusions of the nanofibers.

The presence of α-, β-, and γ-phases was confirmed for both sample types by FTIR and Raman spectroscopy. Based on the FTIR analysis, the content of β-phase was significantly higher for PVDF fibers spun at 2000 rpm, i.e., fibers of thinner diameter.

The never before reported finding is that the higher speed of the collector drum also significantly reduces the wettability as the PVDF fibers spun at 2000 rpm have a higher level of porosity on fiber’s surface than fibers spun at 300 rpm.

The most important conclusion resulting from this study is summarized in the following paragraph. The speed of the collector has a significant influence on nanofibers properties and on the manner of the electrospinning process. This is an obvious fact, but these structures are in this study newly characterized in the most comprehensive way, i.e., (*i*) using all suitable analytical methods and (*ii*) characterizing nanofiber mats as well as the individual (single) nanofiber. This approach presented here, i.e., how to correctly characterize PVDF nanofibers, is recommended to all scientists and should help to better understand and enhance the scientific knowledge about the properties of PVDF nanofibers.

Regarding to piezoelectric analyses, we omitted them in this paper for reason to keep the text clear and easily readable, and the reader is referred to our previous study for these analyses in the introduction part.

## Figures and Tables

**Table 1:** An overview of the processing parameters under which the fibers in this study were spun.

**Emitter**	**Needle size**	**Flow**	**Syringe volume**	**Collector voltage**	**Distance**
Single needle	17 G	18 μL/min	10 mL	50 kV	20 cm
**Collector speed**	**Substrate**	**Temperature**	**Humidity**	**Solution temperature**	
300 rpm and 2000 rpm	Aluminum	24 °C	26 %	60 °C	

## Data Availability

Will be provided at personal request from Nikola Papež. E-mail: papez@vut.cz.

## Sample Availability

Samples of PVDF nanofibers are available on demand from Nikola Papež. E-mail: papez@vut.cz.

## Abbreviations

The following abbreviations are used in this paper:

AC	Acetone
AFM	Atomic force microscopy
DMSO	Dimethylsulphoxide
DSC	Differential scanning calorimetry
FIB	Focused ion beam
FTIR	Fourier-transform infrared spectroscopy
HAADF	High-angle annular dark-field
UHD	Ultrahigh definition
PFM	Piezoresponse force microscopy
PVDF	Polyvinylidene fluoride
SEM	Scanning electron microscopy
STEM	Scanning transmission electron microscopy
TEM	Transmission electron microscopy
XPS	X-ray photoelectron spectroscopy
